# Musculoskeletal Disorder (MSD) Health Data Collection, Personalized Management and Exchange Using Fast Healthcare Interoperability Resources (FHIR)

**DOI:** 10.3390/s24165175

**Published:** 2024-08-10

**Authors:** Fabio A. Seixas-Lopes, Carlos Lopes, Maria Marques, Carlos Agostinho, Ricardo Jardim-Goncalves

**Affiliations:** 1 Centre of Technology and Systems (UNINOVA-CTS) and Associated Lab of Intelligent Systems (LASI), 2829-516 Caparica, Portugal; csl@uninova.pt (C.L.); mcm@uninova.pt (M.M.); ca@uninova.pt (C.A.); rg@uninova.pt (R.J.-G.); 2 Department of Electrical and Computer Engineering, NOVA School of Science and Technology, NOVA University Lisbon, 2829-516 Caparica, Portugal

**Keywords:** FHIR, electronic health records, data collection, internet of things, MSD

## Abstract

With the proliferation and growing complexity of healthcare systems emerges the challenge of implementing scalable and interoperable solutions to seamlessly integrate heterogenous data from sources such as wearables, electronic health records, and patient reports that can provide a comprehensive and personalized view of the patient’s health. Lack of standardization hinders the coordination between systems and stakeholders, impacting continuity of care and patient outcomes. Common musculoskeletal conditions affect people of all ages and can have a significant impact on quality of life. With physical activity and rehabilitation, these conditions can be mitigated, promoting recovery and preventing recurrence. Proper management of patient data allows for clinical decision support, facilitating personalized interventions and a patient-centered approach. Fast Healthcare Interoperability Resources (FHIR) is a widely adopted standard that defines healthcare concepts with the objective of easing information exchange and enabling interoperability throughout the healthcare sector, reducing implementation complexity without losing information integrity. This article explores the literature that reviews the contemporary role of FHIR, approaching its functioning, benefits, and challenges, and presents a methodology for structuring several types of health and wellbeing data, that can be routinely collected as observations and then encapsulated in FHIR resources, to ensure interoperability across systems. These were developed considering health industry standard guidelines, technological specifications, and using the experience gained from the implementation in various study cases, within European health-related research projects, to assess its effectiveness in the exchange of patient data in existing healthcare systems towards improving musculoskeletal disorders (MSDs).

## 1. Introduction

Fast Healthcare Interoperability Resources (FHIR) [1] is an emerging standard that defines common healthcare data concepts and was designed to overcome technical shortcomings of existing standards, leveraging ubiquitous computing and lightweight web services [2]. Introduced in 2011 by the standards international organization Health Level Seven (HL7), it extends and benefits from the knowledge and insights of the implementation of previous specifications (such as HL7 version 2 and version 3) [3] which were standards dedicated to the exchange, integration, sharing, and retrieval of electronic health information. FHIR aims to simplify and enhance this exchange by incorporating the healthcare community expertise and best practices to achieve interoperability between different systems and applications, using a modern and accessible approach.

The building blocks of the standard are resources that provide generic definitions of common health data concepts such as patient, condition, practitioner, observation, and device, at a granular level. The standard leverages RESTful (REpresentational State Transfer) web services and open web technologies for seamless data exchange through APIs (Application Programming Interfaces), positioning itself within the interoperability paradigm and already gaining traction in domains such as mobile health apps, electronic health records (EHRs), precision medicine, wearable devices, big data analytics, and clinical decision support [4]. In 2018, companies participating in the Blue Button 2.0 Developer Conference (Amazon, Google, IBM, Microsoft, Oracle, and Salesforce) announced their commitment to remove barriers for healthcare interoperability, with a focus on cloud and AI-enabled technologies, and signed a pledge to converge and embrace emerging standards such as FHIR for healthcare data interoperability [5]. Most current applications of FHIR are standalone and web-based, implemented across multiple healthcare settings, and are developed by software vendors and mostly targeted at health professionals, rather than patients [6]. A few FHIR application examples are the OneRecord [7], Apple Health Records [8], and Patients Know Best [9]. Nevertheless, the rapid development and adoption of free and easy-to-use FHIR applications in less than a decade demonstrates the momentum and forecasts an accelerated adoption in the future [10].

The FHIR standard is mostly used for clinical data, which is information mostly collected through the traditional healthcare bodies, but wellbeing and physical activity data can also be useful to better understand a person’s health condition and health risk factors [11,12]. Promoting physical activity is valued by health care providers as a promising strategy to improve population health [13], and assessing physical activity through clinical screening tools such as the Physical Activity Vital Sign (PAVS) questionnaires [14], which can be stored in the form of EHRs as shown in the work presented in [12], allows clinicians to evaluate and provide guidance in a primary care setting towards prevention and initial treatment [15], whenever lack of physical activity has predictive validity, contributes to, or is correlated with a certain condition [16,17]. And nowadays, this type of data can be collected by the individual, through their mobile phone apps and wearable devices, that track exercise activity, diet, sleep, and heart rate [18]. The use of these wearables is increasing [19] and using FHIR to structure these data [20] allows for healthcare systems to utilize this vastly collected information to analyze and provide personalized interventions, without handling the heterogeneity of data formats and propriety data storages (from application and wearable providers).

MSDs (musculoskeletal disorders) are impairments of bodily structures such as muscles, joints, tendons, ligaments, nerves, and bones and are correlated with physical activity, working conditions, and perceived quality of life [21], being the most common work-related health problem in the EU [22]. The U.S. Bureau of Labor Statistics estimates that in 2018, 30% of DAFWs (Days Away From Work) are related with MSD cases [23] and in Germany, for example, musculoskeletal disorders accounted for EUR 17.2 billion in loss of production in 2016 and EUR 30.4 billion in loss of gross value added, representing 0.5% and 1.0% of Germany’s GDP, respectively [22]. These disorders must be recognized, considering the different risk factors, symptoms, diagnoses, and treatments, in order to implement prevention plans to reduce its occurrence. To contribute to this, wellbeing, health, and physical activity data, that directly correlate to MSDs, and particularly with low-back pain (LBP) which is a leading cause of disability worldwide [24], can be collected and integrated in EHRs, in an effort to improve decision-making related to prevention and rehabilitation plans and allow for a significant impact on quality of life.

This article explores the contemporary role of FHIR in health-related and wellbeing scenarios, summarizing its functioning, benefits, challenges, and future developments, and presents technical aspects of its implementation through study cases, from European health-related research projects such as Smart4Health [25], SmartBear [26], and ICU4Covid [27], to assess its effectiveness in the exchange of patient data in healthcare systems with a focus on data provided by wearables and medical devices, to target physical activity, wellbeing, and health indicators, in the context of health and rehabilitation for LBP and other MSDs.

The contributions of this article are a data collection methodology that includes a mobile application hub, that integrates wearables and medical devices for acquiring and processing data, and that is integrated into a proposed IoT architecture with specifically designed layers for supporting several functionalities, interoperability across systems, and compliance with existing guidelines. This work proposes novelty FHIR resources that allow for data capture and exchange related to physical activity, posture, wearables, and medical devices in the context of MSDs, with a focus on LBP, contributing to interoperability and decision-making for prevention and rehabilitation plans. Along with these contributions, a literature review on existing applications and the benefits of FHIR is included when relevant, and the output of this work is tested and validated in wellbeing and health-related research projects, with the collection and processing of data pertaining to health, wellbeing, and physical activity.

The structure of this article is as follows. Section 2 describes FHIR history, existing adoption, and the benefits of the standard, and it also explains the resource element and briefly presents other related standards and approaches. Section 3 presents the personalized MSD Health Data Collection and Management solution, explaining the MSD context, the data collection methodology, the developed Citizen Hub mobile application, the interoperable IoT architecture involved, and the implementation of the Continua Design Guidelines. Section 4 presents the proposed MSD Health Data resources. Section 5 delves into three scenarios of data collection and exchange in the context of MSDs for testing and validation of the proposed work. Section 6 presents a discussion of the work and implementation of FHIR. Section 7 presents the work conclusions.

## 2. FHIR

### 2.1. History

The increasing digitization of healthcare information requires data and systems to be interoperable. To standardize this process, Health Level Seven (HL7), a non-profit ANSI-accredited standard development organization, provides standards and promotes health data interoperability. Some previously developed standards are the HL7 version 2 (now out-of-date), HL7 version 3 (lacked wide adoption), and HL7’s Clinical Document Architecture (CDA).

FHIR’s development began in 2011, with Grahame Grieve and a team of HL7 having the idea to combine the best features of previous HL7 versions while leveraging the latest web standards [28]. Originally called “Resources for Healthcare”, HL7’s FHIR has evolved through multiple releases since its initial presentation in 2012.

The first publication occurred in 2013 and went through several iterations before publication as Draft Standard for Trial Use 1 (DTSU1), where it had 49 resources and focused on creating a personal health record on a mobile device where it was possible to retrieve clinical documents. FHIR grew in market acceptance with the publication of the Draft Standard for Trial Use 2 (DSTU2) in 2015, with efforts throughout the Argonaut project [29] where IGs (Implementation Guides) and other means to support adoption by EHR developers and other health IT entities were developed. The FHIR Maturity Model (FMM) was also created, allowing for new resources to be refined and tested before being used in live settings. FHIR Standard for Trial Use 3 (STU3) was released in 2017 with improvements to core clinical, administrative, and financial resources, and developments in clinical decision support and clinical quality measure resources and tools, to allow for easier and faster IG creation and publication to the web. Release 4 (R4) was the first release with normative content in 2019. Two key clinical resources, Patient and Observation, were released as normative, along with the RESTful API, the XML and JSON formats, and nine additional resources [30].

Since then, R4 has been seen as the default option by developers because it is more widely implemented and supported [4], but FHIR Release 5 will see increased normative content [31], with over 30 resources having been nominated. In addition, the community will continue to develop the supportive specifications of FHIR and with the maturation of the FHIR IG tools and templates and better integration with public health, imaging, financial management, genomics, and other fields will keep FHIR at the forefront of the health industry [30].

### 2.2. Adoption and Benefits

The FHIR standard has been mentioned by several organizations when designing and adopting modern healthcare systems. In the EU (European Union), the European Health Data Space (EHDS) recommends FHIR to be adopted as a single health data standard at the European level [32]. The United Kingdom’s National Health Service provides access to national patient identifier systems using FHIR [33]. And in the United States, FHIR APIs are now a federal requirements for EHR certification, according to the 21st Century Cures Act [34] and part of the Policies and Technology for Interoperability regulations of the Centers for Medicare and Medicaid Services (CMS) [35].

For health-related and wellbeing data, there has been some work developed such as [12], where the goal is to use this type of data to assess the performance of physical activity and relate it to a person’s health condition. Apple Health Records [8] is a commercial approach in which this self-tracking data, typically obtained with a mobile phone and wearable sensors, is used to paint a clearer picture of a user’s health and wellbeing. These data can also be integrated from third party healthcare organizations/systems or obtained through manual input or existing EHRs. It includes functionalities to share data with doctors, store records that range from heart rate and lab results to allergies and vaccination, and shows a graphical representation of progress, summaries, and general assessment. This approach follows FHIR specifications, with a focus on SMART (Substitutable Medical Applications, Reusable Technologies) in FHIR (further explained in Section 2.4), while mentioning it as an industry standard, to allow for a seamless integration of data sharing with authorized health organizations and ensuring interoperability with other solutions.

Another emerging application of FHIR is with the International Patient Summary (IPS) [36], which is a standardized document with essential health information, designed to facilitate the exchange of health data across country borders and between healthcare systems, aiming to ensure critical patient information is readily available, especially in emergency situations. IPS benefits from using a standard such as FHIR, to provide a flexible and interoperable framework that is suited for creating and sharing IPS documents, especially considering the need for adoption across borders at a time where huge amount of people migrate to other countries, seeking refuge, employment, or better life abroad [37].

Designed to address some of the challenges and limitations of previous healthcare data exchange standards, FHIR has gained traction as a preferred standard for modern implementations due to several key aspects. FHIR promotes interoperability through a standardized approach for exchanging healthcare data across different systems, applications, and organizations. The data elements, terminologies, coding systems, and formats facilitate a consistent representation and meaningful interpretation of health information, enhancing communication between the different stakeholders [38].

The modular design and resource-oriented approach allow for scalability, while its loose definition of resources, flexible data model, and extensible architecture allow an incremental adoption and integration with existing healthcare systems [39] and evolving requirements and emerging technologies such as mobile apps, wearables, IoT (Internet of Things), big data, and AI (Artificial Intelligence) solutions [40].

The adoption of modern web standards, such as the HTTP (Hypertext Transfer Protocol) and RESTful APIs, and web data formats, such as JSON (JavaScript Object Notation) and XML (Extensible Markup Language), makes it easy to implement and integrate within contemporary system workflows. Its stateless communication model makes interactions simpler, independent, and scalable [41]. In addition to reducing complexity, several Implementation Guides are available to support and clarify the usage in specific scenarios, reducing development effort, allowing new projects to be kickstarted and enabling a faster adoption [42].

FHIR emphasizes a patient-centric approach by focusing on capturing and exchanging patient-specific data [43]. It enables the seamless sharing of patient health records that can support a comprehensive view of the patient’s medical history, supporting informed decision-making and continuity of care [44]. It is designed to be a free non-proprietary system, reducing dependencies and promoting community collaboration in an open-source healthcare ecosystem [45]. It contributes to the continuous development of the standard and the creation of new FHIR applications, tools, and resources.

To provide secure and controlled access to healthcare data, FHIR supports OAuth 2.0, an industry-standard protocol for authentication and authorization, to enable client applications to obtain access to protected resources through credentials [45].

FHIR can be a plug and play standard, despite having loose specifications to provide flexibility, and it is suited for a myriad of implementations in the current technological scenario, with the FHIR resources enabling a modular syntax to exchange data, without requiring the constant adaptation of gateways, through the different communication protocols (such as BLE (Bluetooth Low Energy), MQTT (Message Queuing Telemetry Transport), ZigBee, etc.) and between the multiplicity of heterogeneous sensors, in the necessarily interoperable environment of IoT [46,47]. Based on a report by McKinsey [48], human health and wellbeing represents the second-largest segment/setting in IoT, based on growing economic-value potential, and could reach fourteen percent (with an estimation of between USD 0.5 trillion and USD 1.8 trillion) of the total potential IoT economic value created by 2030. From an infrastructure and software development perspective, the design approach to incorporate web technologies allows for third-party applications to easily connect to EHRs and facilitate processes such as decision-making by seamlessly querying and retrieving data from health information systems with a uniform approach [49].

### 2.3. Resources

**Figure 1:**
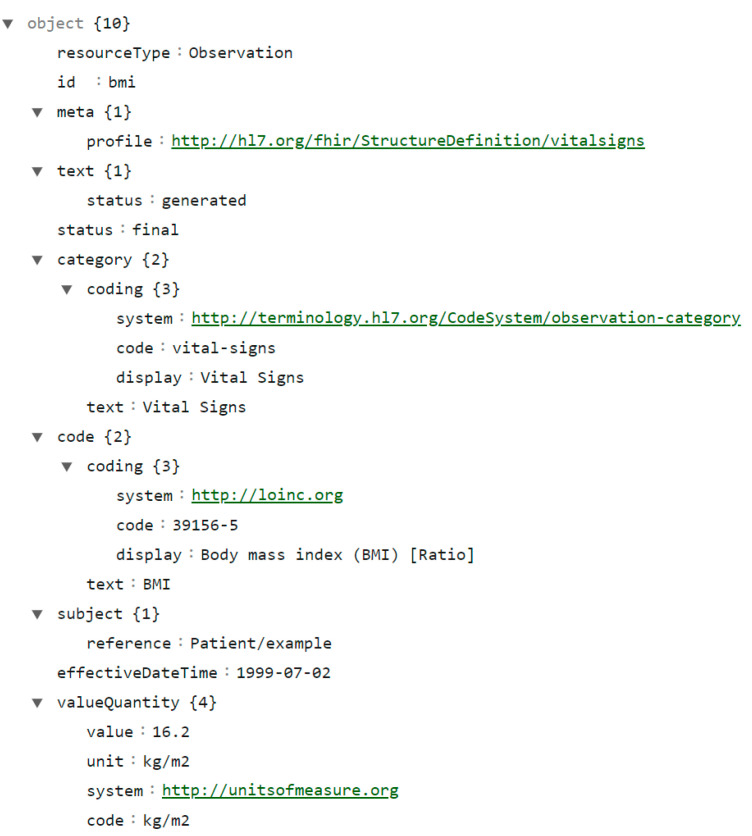
Example of a FHIR resource represented in JSON.

One main objective of utilizing FHIR is to reduce implementation complexity without losing information integrity [4], and the resources are the data elements that allow us to easily describe the activity of healthcare organizations and its stakeholders. To date (in Release 5) there are 158 resources [1] that follow a consistent structure with standard data elements and attributes, envisioned to be modular and reusable. Additionally, FHIR also defines operations, search parameters, and extensions to be used with the resources to provide additional flexibility [50]. A resource, as illustrated in Figure 1, can include metadata, text, or bundled collections of information that form clinical documents. Each resource has a unique identifying (ID) tag, a resource ID, which is used to reference or access a specific resource for various HTTP-based operations such as retrieving, updating, or deleting.

Examples of data elements commonly found in FHIR are the Patient, Observation, Medication, DiagnosticReport, Practitioner, and Encounter resources. FHIR uses JSON and XML structures for data exchange and resource serialization, supporting a RESTful approach that is scalable, loosely coupled, and interoperable.

In Figure 1, an Observation resource is illustrated. It includes coding fields (system, code, and display*)* to represent specific measurement concepts and values through standardized codes or terminologies, to ensure consistency and interoperability throughout systems, such as LOINC (Logical Observation Identifiers Names and Codes) [51], UCUM (Unified Code for Units of Measure) [52], and SNOMED CT (Systematized Nomenclature of Medicine—Clinical Terms) [53]. It defines an example subject (Patient/example) and determines the observation timeframe (effectiveDateTime) and value (valueQuantity), with the corresponding standard unit of measurement (in this example is kg/m^2^), measured during the *Observation*. The measurement itself corresponds to a “*Body mass index (BMI) [Ratio]*” as specified by the LOINC code 39156-5, and is followed the “vital-signs” profile in the HL7 Implementation Guide accessible in [54] which represents physical observations that are an indication of the body’s life-sustaining functions.

### 2.4. Other Standards and Approaches

In the context of healthcare data modeling and management, there are other standards that can be seen as alternatives or complements to the use of FHIR, that generally serve different purposes or have specific objectives. Other standards of HL7 such as HL7 v2 and HL7 v3 are previous standards that rely on more rigid and formal approaches, in comparison with the resource-based structure of FHIR, and require a more complex and expensive implementation [41], which always requires attention to semantic interoperability to ensure that data can be understood across systems. CDA (Clinical Document Architecture) is another HL7 standard that defines the structure for clinical documents for the exchange of patient information, such as prescriptions, medication summaries, and pharmaceutical advice. FHIR resources can be developed to map CDA documents [55], allowing for a combination of these standards that can serve specific use cases that benefit from the richness of clinical information provided by the CDA. A comparison with further description of each of these HL7 standards is provided in Table 1.

IHE (Integrating the Healthcare Enterprise) [57] is a standard that aims to integrate standards and technologies to improve interoperability, by defining profiles, guidelines, and technical frameworks for specific use cases and clinical workflows, and often integrating other standards in its profiles. IHE is a more mature model when compared to FHIR, as it has existed for several years now and is widely recognized and accepted in the healthcare industry, aligning with established clinical practices. Despite that, FHIR offers a more easy-to-use, reusable, and flexible approach, through the resources, with a modern approach based on web technologies [58].

OpenEHR [59] stands for Open Electronic Health Record and is an open standard and framework for the representation, storage, management, and exchange of EHRs and other clinical data. It is designed to be flexible, non-proprietary, and have a standardized structure. It uses archetypes, which are reusable clinical concepts, and templates to model healthcare data, while separating the information layer, which represents data instances or records, and the knowledge layer, which comprises the archetypes and templates. In addition to this, the standard supports the creation of longitudinal health records, which can represent comprehensive patient history over time. Despite that, there is some complexity associated with creating and managing archetypes and templates [60], which may represent a steep learning curve for developers and healthcare professionals.

The Observational Medical Outcomes Partnership (OMOP) [36] is a standardized data model and framework for organizing and analyzing EHRs and observational healthcare data, to support research and clinical studies. This initiative aims to facilitate and optimize research in healthcare, by enabling the conversion of heterogeneous data into a common format, through a created Common Data Model (CDM), facilitating large-scale analytics and observational research. It has enabled research groups around the world to exchange and use observational data for research in a standardized way [61]. It is designed for research and analysis, lacking some versatility and real-time interoperability since it is primarily a data model and not a standard.

SMART on FHIR, while not an alternative to FHIR, is an emerging healthcare application launch framework built on top of FHIR, to tackle the challenge of integrating third-party healthcare applications within EHR systems. The objective is to enable applications to be run unmodified across different healthcare IT systems and it provides built-in components for authorization, authentication, profiles, and UI integration that are not present in FHIR alone [62].

Despite the different approaches and different models, the mentioned healthcare data standards are not directly interchangeable but can coexist through data mapping, transformation, and interoperability tools. Additionally, some of the approaches do not have the same aim as FHIR and are intended for specific use (e.g., research data, in the case of OMOP). For the purpose of this work, none of them directly target wellbeing data, especially considering how data are collected nowadays, using everyday devices, sensors, and wearables. FHIR provides flexibility and has been implemented for the context of lifestyle data in the work presented in [63], which encompasses MSDs and other routine life-impacting conditions, that are influenced by factors such as physical activity, quality of sleep, stress levels, and others. By incorporating these indicators through an effective data collection process (a more comprehensive list of the indicators and the related detailed approach is provided in [64]), it is possible to monitor and manage habits that can greatly impact health and wellbeing, namely MSDs. In the following chapters, an approach to tackle MSD in this context is studied, and a solution that can be personalized is developed and tested, proposing standard FHIR resources for this valuable type of data.

## 3. Personalized MSD Health Data Collection and Management

This work provides the details about the implementation of a solution to monitor and manage MSD health, through leveraging everyday wearable and medical devices, to provide a wider spectrum of data (that can be automatically processed and analyzed) with the objective of painting a clearer picture of an individual’s health and wellbeing.

To accomplish this, it is essential to capture the device measurements through the implementation of a data collection methodology and its integration in an interoperable IoT architecture with specific layers to provide services and other functionalities, while being compliant with industry standard guidelines. With the data being stored and processed, ensuring data quality, they are transferred through the IoT architecture to feed services such as analytics, dashboards, and reports. In this later stage, data follow a medical data standard, FHIR, to ensure that information is interoperable and able to be effectively shared with other health systems and platforms.

**Figure 2:**
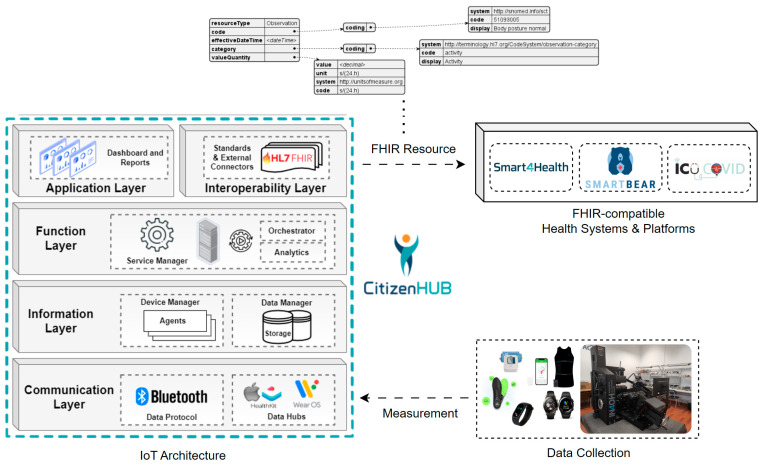
Personalized MSD Health Data Collection and Management overview.

This solution, depicted in Figure 2, is described in the following sections, focusing on the most relevant parts and demonstrating its applicability, in real-world settings.

### 3.1. Context and Novelty of the Approach

Musculoskeletal disorders (MSDs) are soft-tissue injuries caused by sudden or sustained exposure to repetitive motion, force, vibration, or poor posture. These disorders can affect the muscles, nerves, tendons, joints, and cartilage in the upper and lower limbs, neck, and lower back [65]. Musculoskeletal conditions affect people of all ages and can have a significant impact on quality of life. It affects those that are more prone to sedentary lifestyles or suboptimal working conditions, that require sitting for long periods or expose workers to injuries due to incorrect working posture, due to bending, twisting, overreaching, and performing repetitive tasks, which contribute to MSDs [66].

With physical activity and rehabilitation, these conditions can be mitigated, promoting recovery and preventing recurrence [67]. The WHO (World Health Organization) recognizes that a comprehensive response for prevention and control of the main noncommunicable diseases (i.e., cardiovascular diseases, cancer, chronic respiratory diseases, and diabetes) should consider a number of other conditions, such as musculoskeletal [68], which results in reduced work productivity and work absence with a considerable financial impact across healthcare and social support systems. LBP is considered to be the single leading cause of disability worldwide and is increasing in association with a rising and aging population [24], being the condition for which the greatest number of people may benefit from rehabilitation [69]. It is also widely associated with the risk of falls, which can also be associated with cardiovascular and balance disorders, and accounts for most (58%) emergency department attendances in ages over 65, costing Europe over EUR 45 billion by 2050 [70].

Wearables, medical devices, and sensors can provide useful information about a person’s health and wellbeing [71], by providing indicators that allow clinicians to assess and establish a relationship regarding a person’s physical activity. Despite that, to benefit from multiple data sources, a user needs to install different applications where data are presented separately and stored on the manufacturers’ terms. The vendors usually avoid compliance to force users to use their own applications, to avoid sharing these valuable collected data with other companies. Without compliance and the integration of an interoperable system, data owners lack tools to ensure that their data are collected and stored on their own terms, making this an impractical environment.

The proper management of healthcare data allows for better clinical decision support, facilitating personalized interventions and a patient-centered approach. This creates the necessity of solutions and applications that enable the benefits of interoperable standards such as FHIR, within the scope of wearables and personal medical devices, data ownership, and healthcare data exchange.

**Figure 3:**
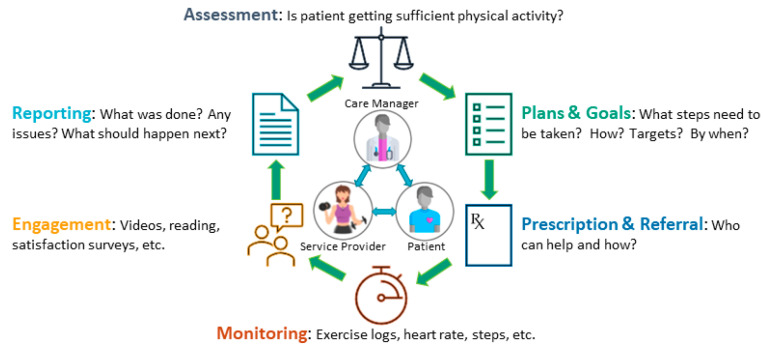
Improving physical activity—healthcare cycle [72].

In this approach, the aim is to describe health-related and wellbeing data, such as steps, calories, heart rate, blood pressure, back posture, and others, to assess a person’s physical state and activity, which, as mentioned before, have a substantial impact on health and specifically on low back health. By structuring and encapsulating measurements of these indicators as FHIR resources, we enable their inclusion in a person’s health record in a standardized manner. A general overview of a possible approach to this is presented in Figure 3, which is specified in the FHIR’s Physical Activity Implementation Guide.

The approach has several steps to assess physical activity (whether using self-tracking data or PAVS questionnaires), plan and establish goals with specific measures and timelines, prescribe and advise to ensure the knowledge of healthcare providers is passed to the individuals, and monitor the more suitable indicators for each case. Beyond that, engagement and reporting allow users to understand the progress in improving physical activity and stimulate further development, by redefining and refining the approach. This cycle requires the care managers, service providers, and patients to be committed and to utilize interoperable standards and reliable sensing equipment that aid in monitoring, assessment, and reporting.

Considering this context and the envisioned novelty solution, as will be presented in the following sections, data are collected from various wearables and medical devices, throughout the whole process of prevention and rehabilitation (which includes medical counseling, exercising, and physiotherapy), that is later integrated into a mobile application that acts as a hub. This application processes and stores data, while providing visual feedback on the collected data. It also enables the user to upload their information to cloud services using FHIR resources in a standardized structure, using novelty resources that leverage physical activity and wellbeing data from everyday use wearables, ensuring interoperability and the further use of this type of information for better clinical decision-making in standard healthcare settings and other compliant authorized applications, allowing for personalized interventions and a patient-centered approach to MSD prevention and rehabilitation plans.

### 3.2. Data Collection

Before capitalizing on the benefits of integrating and analyzing data, the data collection process must be effective and encompass the whole prevention and rehabilitation process, as well as include various sensing devices that cover important indicators of health, daily habits, and physical activity.

At an initial stage, the patient must be examined by a clinical professional, who will accompany their progress, establishing a starting evaluation and, if necessary, a training plan, whether it is for prevention or rehabilitation purposes. Also, it provides some additional recommendations for the patient’s habits and lifestyle, that can improve health and wellbeing indicators.

**Figure 4:**
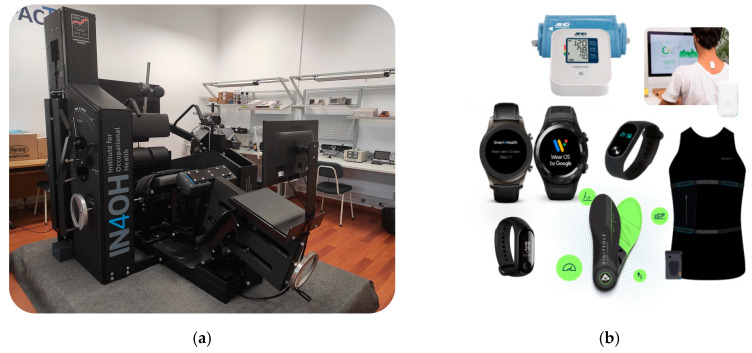
Hardware used in the data collection: (**a**) MedX lumbar extension machine; (**b**) wearable devices.

In the context of this work, considering the protocol of the implemented solution, and after the clinical evaluation, the patients that are subject to physical therapy training are accompanied by physical therapists for several weeks and perform gamified lumbar training exercises on a MedX lumbar extension machine [73] (Figure 4a), that directly improve the strength and motion of the patient lumbar musculature. The results of the training and the collected measures are uploaded for further analysis and integrated with data from other devices. These devices (Figure 4b) are wearables and medical personal devices that measure important health and wellbeing indicators without major inconvenience, and that are able to communicate and easily be integrated with the patient’s phone (using a standard communication protocol such as Bluetooth).

Devices currently compatible with the solution and the mobile application presented in the next subsection are selected fitness bands, posture sensors, smartwatches, smart insoles, smart vests, and blood pressure monitors. Some collected metrics are the number of daily steps, distance walked, pace, burned calories, current heart rate, evaluation of back posture, breathing rate, stride quality, and others.

With this information, the personalized solution for MSD gains shape, but the data need to be collected, filtered, analyzed, and displayed. By processing and aggregating information, it is possible to paint a clearer picture of a certain person’s health and wellbeing, serving as a complementary tool for health assessment and diagnosis, while establishing a measure of progress that can be integrated to personalize the prevention or rehabilitation training process.

### 3.3. Citizen Hub

To effectively collect and process these data, an interoperable solution must be implemented in an IoT paradigm that supports the existing heterogeneity of sensing devices and applies methodologies to ensure data are usefully handled. Citizen Hub is a new mobile digital health solution, where the user chooses how data are collected and stored. It emerged as a gateway application and integrates several wearables and medical devices, providing features that allow for monitoring and managing habits that can greatly impact health and wellbeing. Since collecting data is a continuous process, several elements for communication must remain active and deal with the heterogenous environment of wearables’ behavior, protocols, and messages of communication.

**Figure 5:**
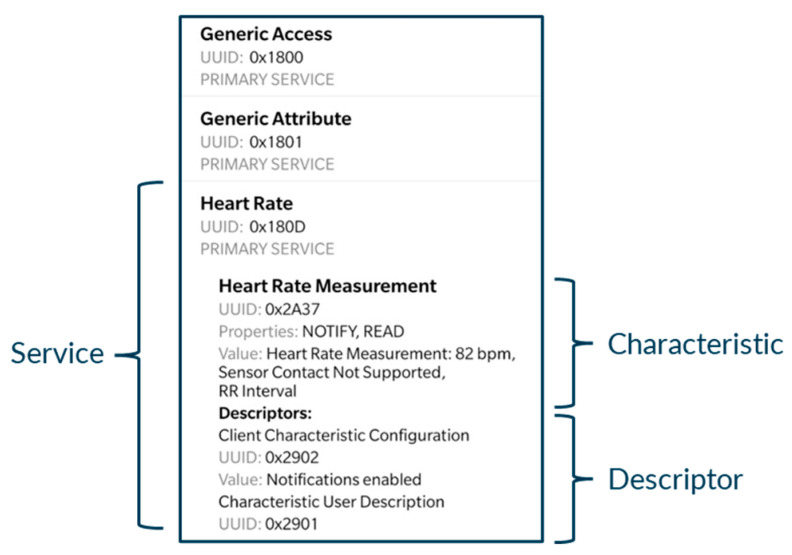
BLE device attributes’ structure example.

The BLE (Bluetooth Low Energy) protocol is the most adopted standard communication protocol for wearables [17]. It specifies different layers for communication between devices that ease processes such as advertising, scanning, and connecting. Within the BLE context, a *service* is one or more attributes that specify a general functionality of a device (which can have many). A *characteristic* is a part of that service and defines specific information provided within that service. Those services and characteristics have a specific corresponding standard *UUID* (Universally Unique Identifier), as shown in Figure 5 (e.g., if a device advertises the service *UUID* specific for heart rate “*0x180D*”, other devices may access that service already knowing it includes heart rate measurements, where the characteristic with *UUID* “*0x2A37*” provides the heart rate measurement values). Additionally, there is a descriptor that can be used to manage the communication, showing if notifications or indications are enabled.

Within Citizen Hub, if devices are compliant with the BLE standards, integration is a simple process, where the services and characteristics are understandable, and the methodology can be reused for devices of the same type. If not, and since many devices output more than one type of data and have multiple services and characteristics, it can be very time consuming to try to understand what values correspond to what measures, or even understand what the device really measures at all. To overcome this, reverse-engineering processes may be needed to understand how each of these devices works and how to effectively integrate it.

Reverse-engineering is a methodology to discover and define an object’s functioning [64] by various methods such as specific testing, analysis, and comparation. For this specific case, it is important to understand how the device communicates to allow interaction. By repeatedly making certain movements or forcing certain outcomes, sometimes using other similar devices to compare measurements, it was possible to decode communication patterns and isolate certain device behaviors. After thoroughly analyzing this information, which sometimes corresponded to thousands of communication packets per minute, several device services and characteristics were successfully attributed to functionalities. Despite the effort, for devices that do not follow BLE standards, integration was not possible or some secondary features were not able to be fully integrated, sometimes presenting inconsistent behavior during long-term functioning.

**Figure 6:**
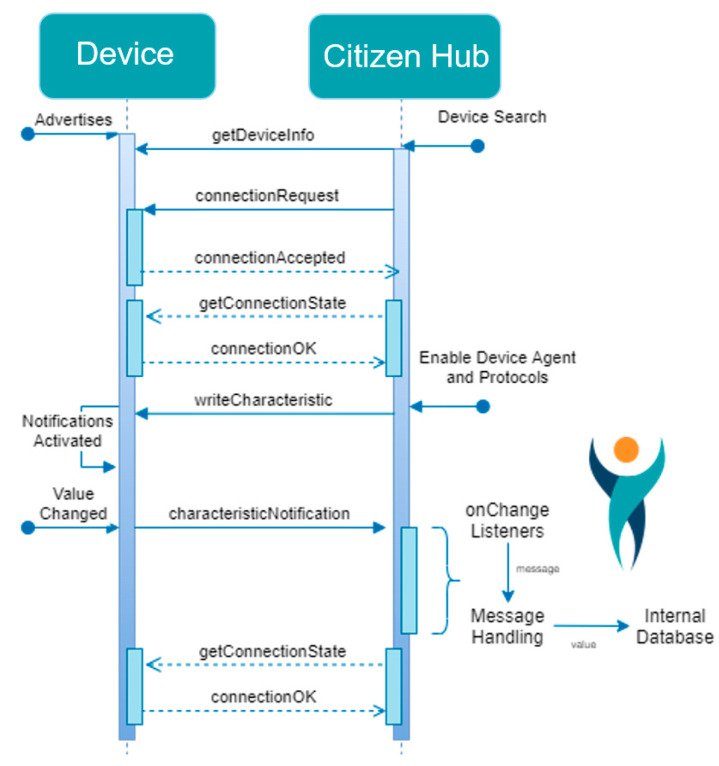
Communication between the device and Citizen Hub [74].

But once it is clarified how the device provides information and what the information means, communication can be established, as illustrated in Figure 6.

A continuously running background service is used to create and manage communication-handling agents that deal with each connection to devices. These agents contain device information, features, protocols, and any additional algorithms to filter and process the data. Each agent lasts the same time as the corresponding connection, and the objective is to establish a bridge of data between the device and the hub.

Initially, the Citizen Hub application searches for available devices and displays a list to the user. The user then selects a device and a connection is established. After that, the device notifies the user whenever new data are collected and sends the information. Citizen Hub receives and processes the message, storing the result internally. The integration process is described in more detail in [74]. Then, Citizen Hub is able to display real-time and aggregated information to the user, based on the collected data, and that information can be uploaded to a cloud platform or sent to other services or third-party applications. 

**Figure 7:**
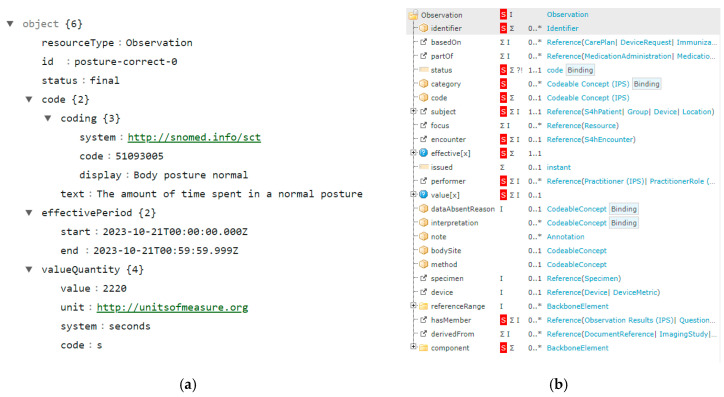
Observation definition in Citizen Hub: (**a**) observation resource example for a posture measurement; (**b**) observation resource specification.

To ensure interoperability, data are structured to be compliant with FHIR, which will be explained in more detail in the next section. In this type of structure, Citizen Hub defines each measurement as an Observation as represented in Figure 7, with Figure 7a showing an example of a posture observation over a specific timeframe and Figure 7b illustrating the resource specification.

The data provided in this Observation are bounded to a specific timeframe (i.e., when the measurement occurred), to a context (to further analyze the validity of the data), and are associated with a coding system (referring to existing reference terminologies and formats), so other systems can identify and meaningfully process the data provided.

### 3.4. IoT Architecture

**Figure 8:**
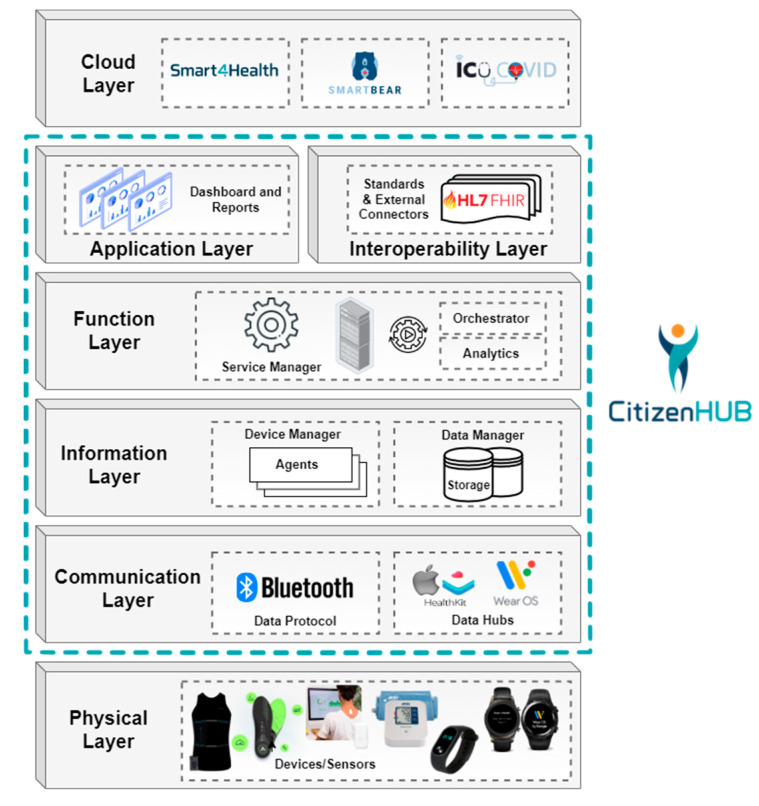
Citizen Hub IoT architecture.

To ensure that Citizen Hub performs an effective data collection and data are effectively processed, stored, represented, and made available in a useful approach, an **IoT architecture**, based on commonly used layered IoT architecture approaches as studied in [75], was envisioned to guarantee proper interoperability and functioning in the context of this work. Within the designed architecture, five specific layers are encompassed by the application, which connect to two other layers to collect and to provide data to, as illustrated in Figure 8.

The IoT paradigm was first defined by the means of computers being able to access data about objects and the environment without human intervention [76]. By connecting and providing communication abilities to sensors, sensing networks emerge as a valuable solution that aim to connect everything to the Internet. In this way, the information gathered by data collection can be processed, stored, and used for decision-making within complex systems that have a large number of variables and require real-time or readily available data from its assets, processes, and environment [77].

The created architecture defines different layers that fulfill the needs and functionalities of the Citizen Hub application, with the lower layers following an ETL (Extract, Transform, Load) data ingestion approach [78] and the upper layers being designed for user interaction, compatibility, interoperability, and services/functionalities. 

A brief explanation of the different layers of the architecture is provided below, expanding the work presented in [64].

***Physical Layer—***Includes the various data sources (medical devices, wearables, and others) that were integrated and successfully communicate with the mobile application using standard protocols. The selected data sources are mostly wearables and devices that aim to target physical activity, wellbeing and health indicators, and the data from the clinics and the respective lumbar physiotherapy machines, with the objective of retrieving, processing, aggregating, and summarizing the available health and wellbeing information about the user.

***Communication Layer—***Handles communication with the devices from the physical layer, supports the different standard data communication protocols, applies pre-filtering techniques to ensure data quality, and performs communication related to requests from upper layers.

***Information Layer—***Processes the incoming data to make them usable. The Device Manager contains the data models and properties of the connected devices, which are handled with connection agents. The Data Manager uses protocols to filter, transform, and save the information into internal storage.

***Function Layer—***The function layer represents the remaining backend work after the data are consolidated. The Service Manager contains continuous services that handle the general operations of the application, managing workloads and the backstage for application layer tasks.

***Application Layer—***This layer holds the different services and functionalities that are provided to the user through graphical interfaces. It includes dashboards for configuration, monitorization, integration, and any supported analysis of data. It also includes other available services, provided by the work of other architecture layers or authorized third-party applications.

***Interoperability Layer—***Allows for communication with external systems through standards such as FHIR and contains dedicated connectors to cloud services using specific SDKs (Software Development Kits). This allows for the easy adaptation, integration, and sharing of data across different systems.

***Cloud Layer—***This layer represents cloud infrastructures that are interoperable and compatible with the used standards. This layer is the recipient of the information that the user chooses to upload and provides access to additional processing, services, and functionalities. In this architecture, the platforms depicted are related to the testing and validation of this solution in the context of EU health research projects, which will be explained further in this work.

With data being uploaded to the cloud platform in an interoperable and sharable format across systems, information from the wearable devices and from the MedX lumbar extension training machines, from several physiotherapy clinic sites, is able to flow and be processed throughout the different layers of this IoT architecture. In this way, it allows for the development of services and functionalities that empower decision-making and allow users to achieve personalized insights, that are helpful for the user and the rehabilitation training process.

The platforms presented in the cloud layer are related to EU health and wellbeing research projects (that will be further explained), that successfully implemented the solution described in this work and that deal with a significant number of datasets of device measurements, EHRs, medical data, and related information. These platforms are capable of ingesting FHIR structured data and were used to validate the cross-platform functionality of this solution.

### 3.5. Continua Design Guidelines

To ensure that the architecture communication and data collection processes are seamless and compatible with contemporary common devices, the architecture must envision the integration of devices that are effectively compliant with communication protocols guidelines and industry standards. The Personal Connected Health Alliance (PCHA) publishes and promotes the global adoption of open standards and guidelines, ensuring conformity of medical-grade data exchanged between sensors, gateways, and end-services, with the objective of making implementations truly interoperable, beyond the envisioned reference standards [79].

**Figure 9:**
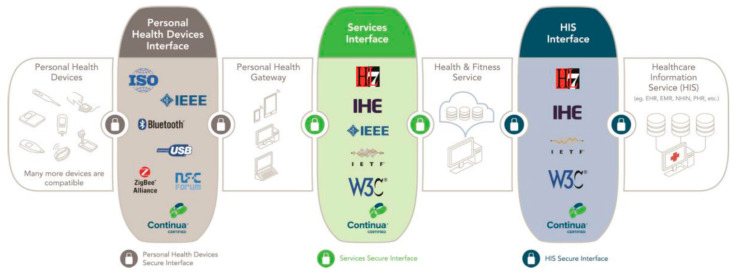
Continua end-to-end reference architecture [80].

The Continua Design Guidelines (CDG) is an open framework, promoted by PCHA, for interoperable data exchange in personal connected health, providing clearly defined interfaces that enable the secure flow of medical data, removing ambiguity in underlying standards, to ensure a consistent and interoperable ecosystem of personal connected health devices [80]. The Continua end-to-end reference architecture is shown in Figure 9 and serves as a guide to design and certify the different components of health-related systems.

Considering the beforementioned issue, regarding the compliance of the BLE devices, by ensuring adherence with FHIR, Bluetooth, and other standards, compatibility beyond CDG is assured. For the devices, PCHA defines the Personal Health Devices Interface that determines a framework of standards and criteria, that aims to achieve the interoperability of devices and data used for personal connected health services [80]. The integration of these devices is considerably easy when compared with non-standard devices, since device behavior is expected and services/functionalities are clear and easy to access, interact, and integrate.

**Figure 10:**
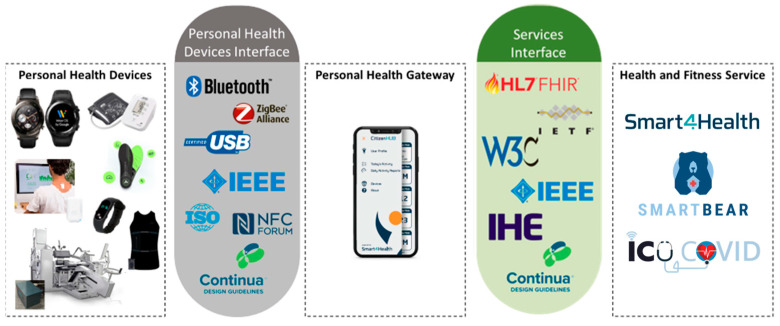
Implementation of CDG in Citizen Hub, based on the reference architecture.

The result of the implementation of this reference architecture and the different Continua components is illustrated in Figure 10, where Citizen Hub acts as a Personal Health Gateway and the cloud platforms are part of the Health and Fitness Services.

## 4. MSD Health Data Resources

For the adoption of FHIR, within the context described in this work, the Citizen Hub application was created and can be configured to relay the collected wearable data to a cloud platform, using a platform SDK, which acts as a connector. In its current version, Citizen Hub is connected to the Smart4Health cloud platform, but similar implementations were developed for other health, wellbeing, and rehabilitation projects (SmartBear and ICU4Covid), demonstrating the cross-platform capabilities when an application follows an interoperable IoT architecture and widely adopted standards.

**Figure 11:**
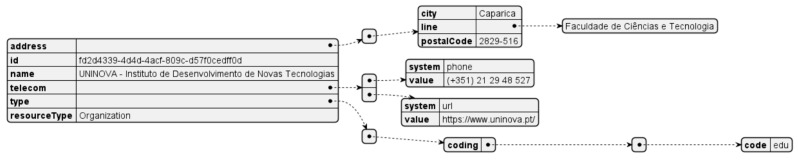
Resource for authorship of data.

Citizen Hub uploads the collected data in two different ways, depending on its target use case. Binary files in the form of PDF (Portable Document Format) documents are uploaded to the platform to provide a resource that is easily readable by the user and FHIR resources are uploaded containing the same information targeting systems and machine processes. Both these types of resources are based on FHIR, where the binary files are wrapped as an attachment in a DocumentReference type of resource. The resource shown in Figure 11 is used to mark the authorship of the communicated data, when uploading other resources.

The user is able to configure what data are meant to be uploaded to the platform and which are not. The reported data exist in the two beforementioned versions, the machine-readable version, consisting of FHIR resources, and the user-readable version, consisting of a visual representation in PDF format.

**Figure 12:**
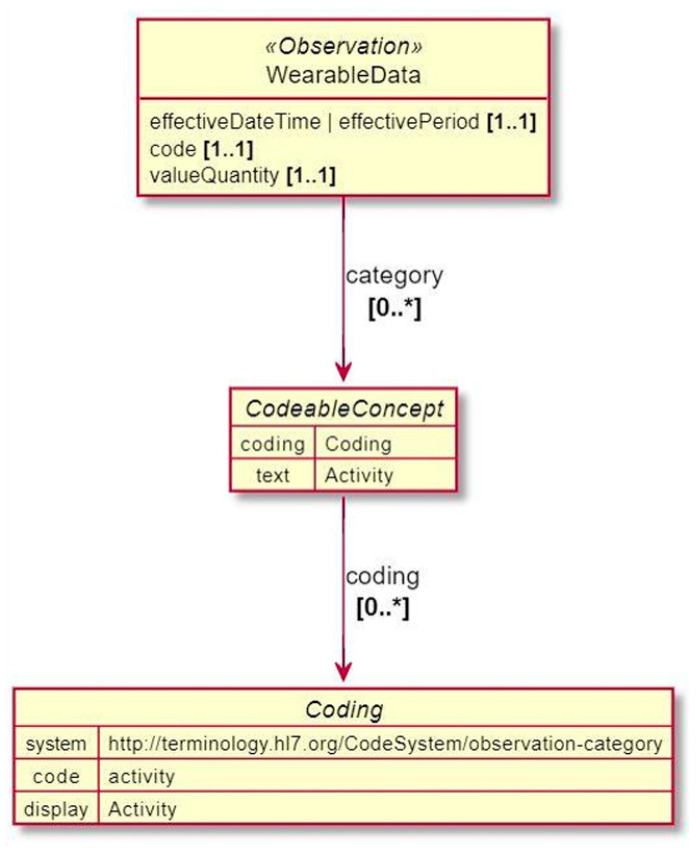
Wearable data profile [81].

For the purpose of representing the collected data and reporting to the platform in an exchangeable format, and following the FHIR principles, the collected measurements and health/wellbeing indicators are represented as observations following the wearable data profile depicted in Figure 12. These observations have an associated timeframe and coding system to ensure context and interoperability across different systems. A detailed explanation for how wearable data are encapsulated within an observation can be found in [81]. The structure for these observations is presented through example resources in the following sections.

### 4.1. Heart Rate

**Figure 13:**
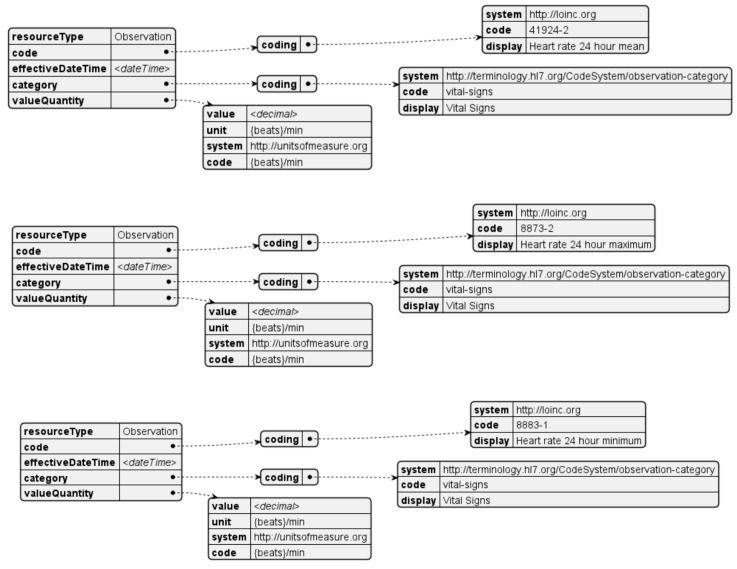
Heart rate observation resource.

Measurements of heart rate are taken throughout the day and aggregated in three metrics over a twenty-four-hour period: mean, maximum, and minimum. These metrics are presented in the standard unit for heart rate measurements, beats per minute. The corresponding FHIR resources are generated for every day for each of the mentioned metrics. The template for this resource is depicted in Figure 13.

### 4.2. Activity

**Figure 14:**
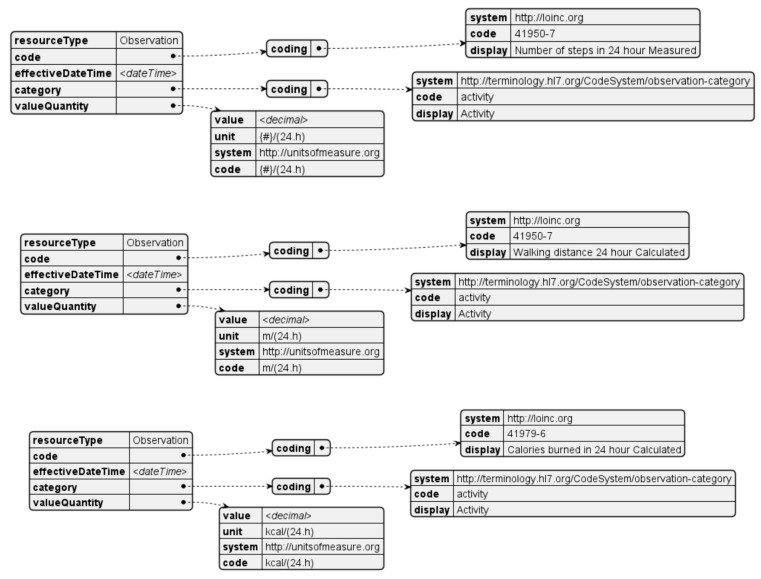
Activity observation resource.

Activity time measurements (Figure 14) are taken throughout the day and aggregated in three metrics over a twenty-four-hour period: steps, distance, and calories. Steps are typically measured with a pedometer and are represented as the number of steps per day. Distance and calories are usually calculated with the original step count or through more advanced algorithms (depending on the device). These are represented in meters and kilocalories, respectively.

### 4.3. Posture

**Figure 15:**
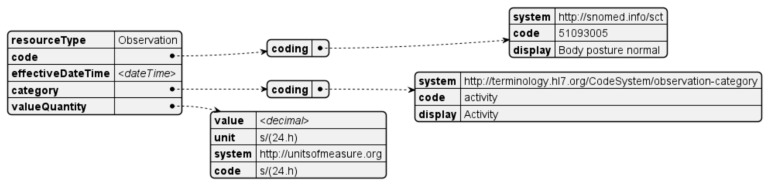

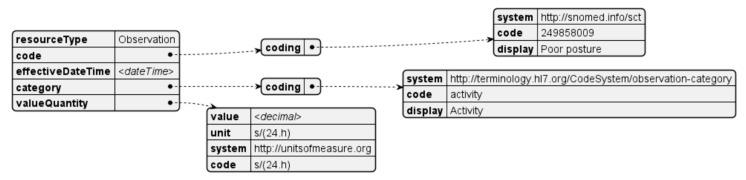
Posture observation resource.

Posture measurements are taken throughout the day and are classified according to pre-defined posture angles and considering an initial calibration process, that determines normal and poor posture. The resulting values are reported in seconds over a twenty-four-hour period. The corresponding resource is generated every day for each posture classification (Figure 15).

### 4.4. Blood Pressure

**Figure 16:**
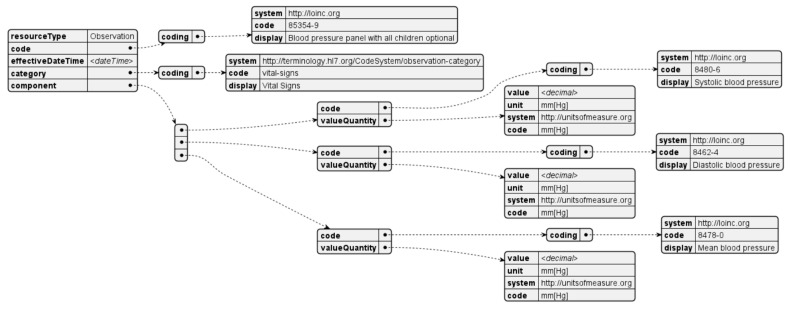
Blood pressure observation resource.

Blood pressure is measured on demand and using compatible devices. Each measurement is comprised of systolic pressure, diastolic pressure, and mean arterial pressure, represented in millimeters of mercury. An FHIR resource is generated for each measurement taken (Figure 16).

### 4.5. Lumbar Extension Training

**Figure 17:**
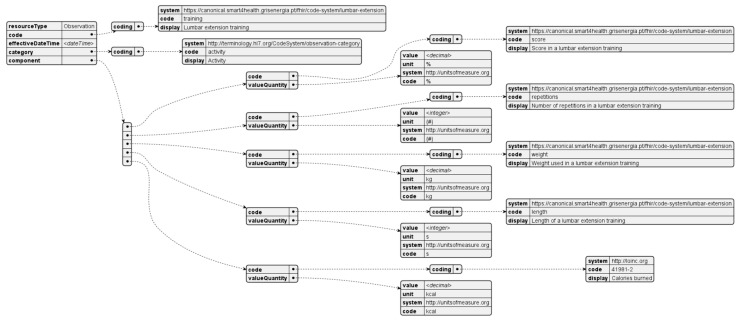
Lumbar extension training observation resource.

The lumbar extension training is a result of performing a low back rehabilitation session in a MedX lumbar physiotherapy machine. After the session, the patient can connect to the machine and use Citizen Hub to fetch the results of the training. For each session, a FHIR resource is generated as shown in Figure 17.

### 4.6. User Reports

**Figure 18:**
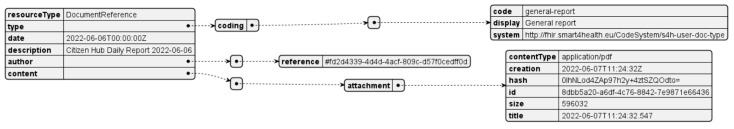
Report resource.

The resources created for user readability are presented as reports. These reports are generated daily for aggregated data and once off for discrete measurements. Each of the measurements described before can be a component of a constructed report. The basic *DocumentReference* is transversal to every report uploaded. An example is provided in Figure 18.

### 4.7. Unique Device Identification

**Figure 19:**
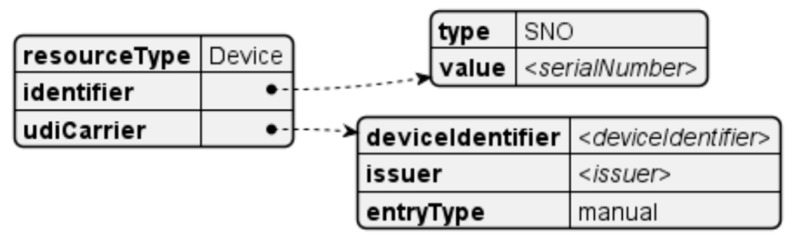
Device resource.

When the user defines a configuration setting for a device, that information is attached to each observation resource sent. This relies on the user entering the serial number of the device along with the device identification. With that information, the mapping described in [82] is performed. The FHIR template for the *Device* resource is depicted in Figure 19.

### 4.8. Validating Resources

**Figure 20:**
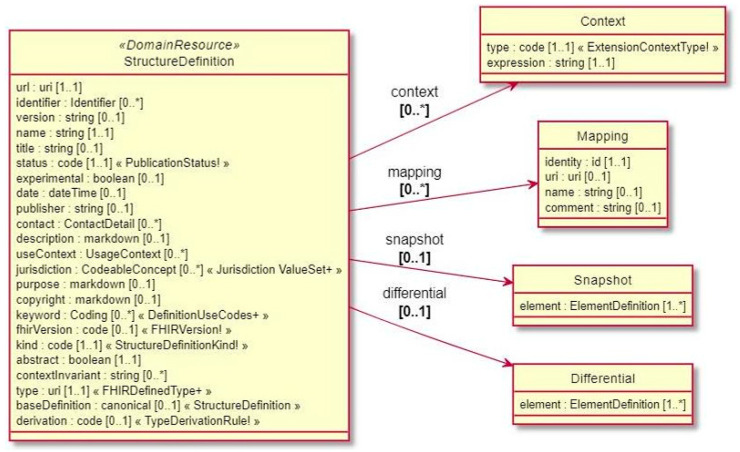
Structure Definition data model.

The FHIR specification delineates a collection of fundamental resources, frameworks, and APIs employed across various healthcare settings. Nevertheless, disparities in practices, requirements, regulations, and other aspects exist across different jurisdictions and within the healthcare ecosystem. This is why FHIR serves as a platform specification, establishing a shared foundation upon which a multitude of diverse solutions can be built and implemented. Therefore, this specification requires further adaptation to contexts of use. FHIR defines a cascade of artifacts for this purpose. IGs define a wider scope and represent a coherent and bounded set of adaptations, and validation occurs within the context of each IG. A profile is a set of constraints and extensions for a certain resource, represented as a structure definition, and can be a mechanism to customize and specialize FHIR resources to meet the requirements of specific use cases. By extending and restricting a resource (or *profiling a resource*), it is possible to limit or specify allowable values, cardinality, and other relevant characteristics of elements within FHIR resources, which is useful for ensuring that the data conform to particular standards or requirements, and include additional elements or extensions to capture specific information. Profiles can be shared and reused across different implementations, enabling consistency and interoperability in diverse healthcare settings. This is typically defined with a *Structure Definition* resource, illustrated in Figure 20, which serves the purposes of profiling and aligning resource definitions, thus facilitating validation across systems and reducing ambiguity. Additionally, validators such as [83] are useful tools to verify if the different aspects (structure, cardinality, profiles, etc.) of a resource are valid within the specification. This verification can be made automatically by applications in healthcare systems, when receiving or sending content.

## 5. Demonstration of Results in Real-World Settings

The previously presented resources were used in the context of EU research projects for health, rehabilitation, and wellbeing. This allowed researchers to design, develop, and test the implementation presented, while assessing the capabilities and limitations in different scenarios, with multiple users, several types of devices, and data. This also validates the versatility and maturity of the whole solution for different settings and purposes within the eHealth framework.

### 5.1. Health and Wellbeing Data Collection towards Back Pain Prevention and Rehabilitation

The Smart4Health [25] project is a Horizon 2020 European initiative aimed at developing a citizen-centered EU-EHR exchange platform for personalized health, enabling citizens to manage and bridge their own health data throughout the EU and beyond, allowing users to store, manage, and share data securely with professionals and trusted ones, with access to personalized insights. With the smarterization of physiotherapy machines and the integration of wearables’ data in Citizen Hub, the use cases were focused on the citizen’s health in daily life, work, travel, and in healthcare settings, in several locations but with the most expression on the island of Madeira. The project involved hundreds of participants recruited from various demographics over 18 years old, that translated into several data sets collected. Participants were able to take part in physical rehabilitation sessions with trained therapists and connect their wearables, medical devices, and other compatible equipment to the Citizen Hub mobile application, uploading data to the Smart4Health cloud platform to take advantage of the project’s services and functionalities. In total, considering eight use case scenarios, around 135,000 data sets were collected that included the rehabilitation physiotherapy training sessions and indicators such as steps, calories, distance travelled, heart rate, blood pressure, back posture, and other physical activity and health related measurements. The data shown in Table 2 are separated by use case, in this project with the designation of CUCs (Citizen Use Cases), where each focused on different aspects of the project intervention. Each CUC addressed high-relevance societal and economic health issues through the different stages of diagnostics, treatment, prevention, occupational health, and practical involvement with the objective of connecting real-life setting examples of enabled citizens that manage, collect, access, and share their own health and healthcare data [84].

Smart4Health provides the possibility of sharing this wellbeing and health data with different clinicians, medical centers, and local and international organizations for research purposes, as well as to engage directly with healthcare providers. The users are able to upload data to the cloud platform, using EHRs and other mentioned sources and formats, from their mobile application or the project’s web portal, accessible at any time or anywhere. This data are shareable with authorized healthcare providers in critical emergency situations or with other people of trust, such as a designated relative.

**Figure 21:**
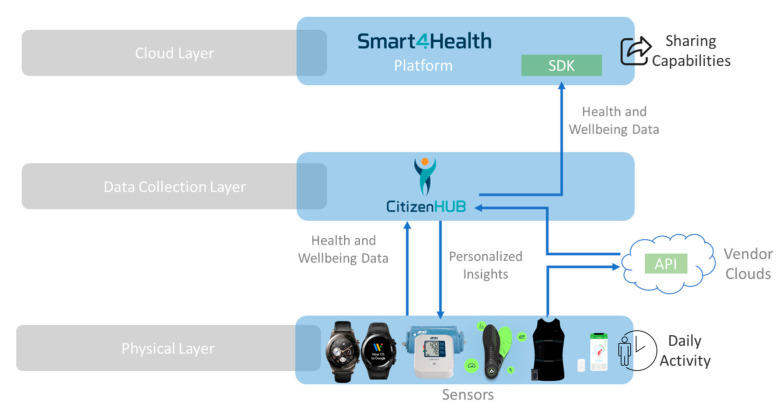
Citizen Hub data collection simplified architecture.

In Figure 21, a simplified view of the data collection architecture is illustrated (implementing the IoT architecture shown in this work), where data flows from the physical layer, which encompasses the compatible devices, to the Citizen Hub application, where the data are processed, stored, and personalized insights are generated.

This information may be transferred directly through Bluetooth or through APIs of third-party/vendor clouds to ease the communication process. When and if the user chooses to upload the information to the cloud platform, an SDK connector allows for a seamless data transfer in FHIR format, using the previously presented FHIR resources. The data stored in the platform can be later shared with authorized clinicians or any other trusted entity, with the objective of enhancing decision-making in back pain prevention and rehabilitation, in this way targeting MSDs.

### 5.2. Data Collection for Personalized Healthcare for the Elderly

SmartBear [26] is an ongoing EU research project that aims to seamlessly integrate wearables’ data, sensing IoT devices, and assistive medical and mobile devices to enable continuous and objective data collection from the everyday life of the elderly. It has the objective of providing personalized healthcare that aims to monitor and improve the individual’s health condition, while also preventing illnesses. Some targeted illnesses are cardiovascular diseases, mood disorders, balance disorders, hearing loss, cognitive disorders, and frailty, which are prevalent conditions with tremendous social and financial impact.

The project is ongoing and already has recruited over 100 participants with different backgrounds, representative of the elderly population, in a Pilot of Pilots (PoP) initiative within the Madeira island, where participant kits were distributed with a mobile phone device, personal devices (medical and wearables), and smart home devices, that will allow users to collect several data sets for physical activity assessment such as blood pressure, heart rate, back posture, steps, calories, weight, sleep quality, and others. Participants were also able to participate in low back rehabilitation sessions with physical therapists, participate in balance rehabilitation sessions, and connect their wearables to Citizen Hub and the SmartBear platform.

So far, around 3 million datasets have been collected and transferred to the SmartBear platform and 2000 to the Smart4Health platform, with the MSD relevant data being structured and exchanged using the FHIR resources proposed in this work.

**Figure 22:**
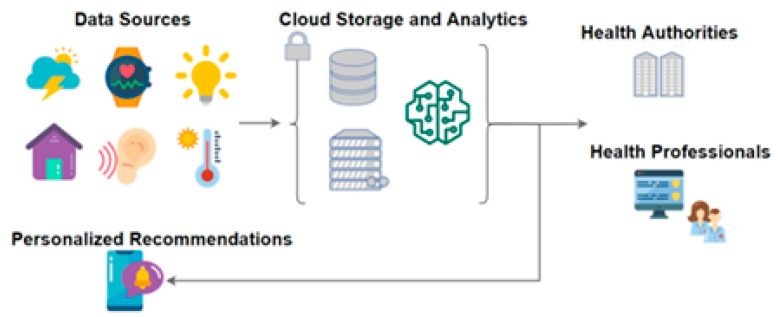
Citizen Hub data collection simplified architecture [85].

The simplified architecture of the SmartBear projected is depicted in Figure 22, with a similar approach to Smart4Health (using the IoT architecture proposed in this work), where data are collected, processed, and stored, providing insights and personalized recommendations.

Through this synergy, between the SmartBear and the Smart4Health projects, which conducted pilots in the same geographical location and both targeted low-back pain conditions, with tools and data being shared between the projects, a sustainable framework for wellbeing is being tested and validated.

**Figure 23:**
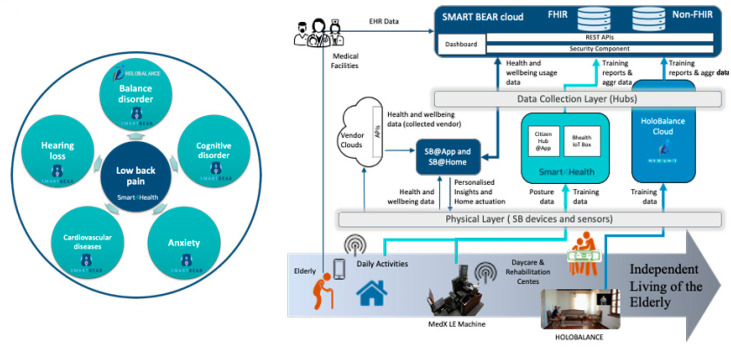
Comorbidities addressed in the PoP and technical overview for data sharing [86].

In Figure 23, an overview of the comorbidities addressed is shown and a technical overview for data sharing is provided, depicting the data flow and the relation between the conditions and the gathered information.

### 5.3. Wearable and Environmental Data Collection in ICU Settings

**Figure 24:**
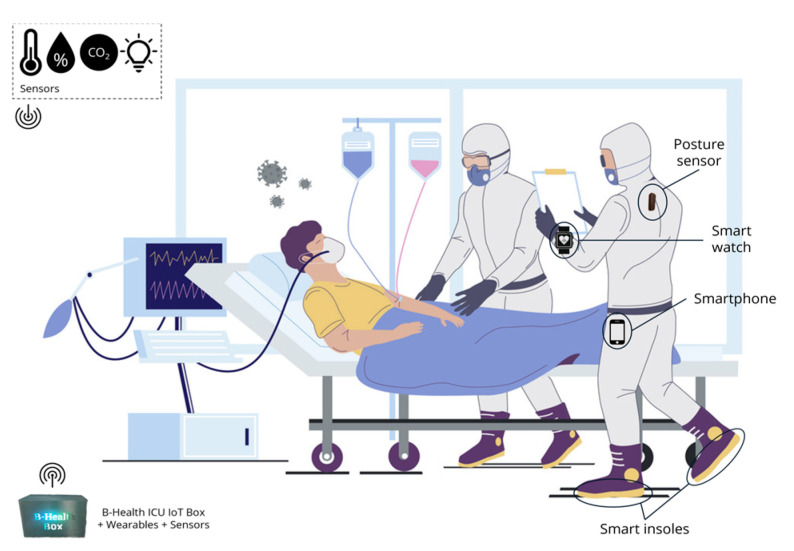
ICU4Covid implementation of devices and sensors [27].

ICU4Covid [27] is an EU research project that aimed to develop a cyber-physical system called CPS4TIC (Cyber-Physical System for Telemedicine and Intensive Care) at a large scale, delivering intensive care medicine fit for the fight against COVID-19 and further outbreaks or similar diseases. The system consists of a telemedicine cockpit, a connector platform, and smart bedside hubs (depicted in Figure 24), that enabled continuous real-time monitoring and a smart care environment.

The project targeted the prevention of infection for the health workforce and patients suffering from this condition. Over 25 healthcare providers located in various European countries were able to use wearables integrated with Citizen Hub for continuous monitoring. Other indicators were collected using the BHealth ICU IoT Box, a hardware that implemented environmental sensors and followed the IoT architecture presented in this work (acting as a hub). Data sets were collected and exchanged, regarding indicators such as posture, steps, calories, heart rate, and other related measurements. Environmental sensors (i.e., CO_2_, temperature, humidity, luminosity) were also implemented in the ICU (Intensive Care Unit) of four hospitals, to provide data for the hospital authorized personnel to contribute to a safer environment.

## 6. Discussion

### Challenges and Future Developments

Despite the benefits of adopting the FHIR standard, a few challenges are still associated with its implementation. FHIR allows for a flexible interpretation of profiles and a standard structure, which can lead to variability beyond interoperability, making it challenging for different systems to communicate, this way requiring additional mapping and transformation efforts to ensure data compatibility [82]. Pre-requirements could also hinder its implementation, both from an infrastructure and personnel perspective. Systems need to be adapted and capable of storing and managing data in the context of FHIR, possibly needing complex, extensive mapping and data transformation processes to retrofit legacy functionalities and data formats [87]. The healthcare professionals need to be equipped with the necessary knowledge and skills to effectively use FHIR, changing existing workflows and processes to incorporate the standard without disrupting clinical operations [88]. Data quality is also a concern, because inconsistencies, missing or incomplete information, or variations in the data formats can limit the implementation and potential interoperability [89]. Healthcare data contain personal and sensitive information, so ensuring data security, integrity, confidentiality, and privacy is critical. Additional robust security measures for authentication, authorization, logging, and auditing mechanisms need to be considered, validated, and documented for general use [90]. Lastly, FHIR needs to be able to handle and adapt to emerging technologies and the increasing volume of data that comes with them, so sustainable development is also critical to ensure the successful future and adoption of the standard.

From the mobile application perspective, there were technical challenges regarding the integration of devices, due to most of them not being compliant with the BLE standard and requiring a considerable effort to associate services and characteristics to the features. Additionally, the inconsistency of the BLE devices required additional mechanisms to deal with communication, reconnection, and synchronization. The mobile operating systems also provided some issues while conducting their battery and process optimization procedures, which varies for each mobile manufacturer, requiring additional testing and access to several different mobile devices.

To identify these issues, multiple tests were performed to validate the approach, such as connecting different devices with different configurations and testing their limitations during their performance, forcing them to communicate with other applications to understand their modus operandi, and testing the overall limits of the BLE protocol through reverse-engineering processes.

**Figure 25:**
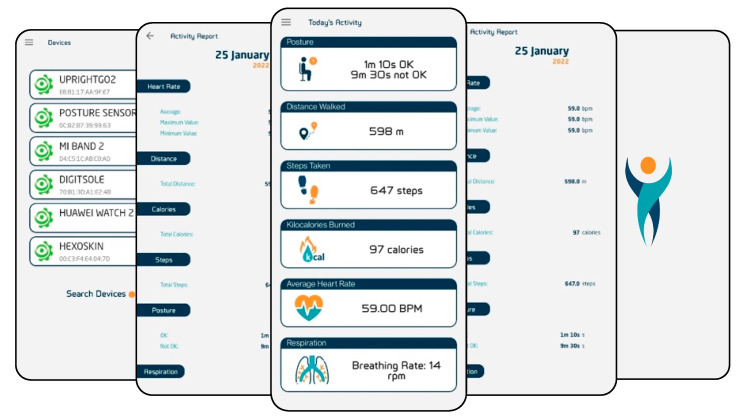
Citizen Hub user interface.

Citizen Hub’s user interface is illustrated in Figure 25 and displays some of its functionalities such as managing devices, viewing activity reports, and displaying real-time data.

Citizen Hub is already a fully functionable application that integrates various devices and is free to use, without the need to register with any of the project platforms presented. It utilizes the provided data collection methodology while being integrated in the proposed interoperable IoT architecture with specific layers to provide the described functionalities and be compliant with necessary guidelines.

The proposed MSD health data resources were developed and revised after their implementation in the previously mentioned scenarios, where data related to MSDs were collected and encapsulated in these resources to be readily available for exchange with other systems in the FHIR format.

In the future, the FHIR implementation will be further explored to ensure further interoperability with third-party systems, and there will be new functionalities and a higher level of detail for the resources used (possibly adding new resource types as technology evolves and new devices measure other relevant indicators). Also, it is important to add compatibility with more devices for the Citizen Hub application, while providing continuous updates for the new firmware versions of already implemented devices.

## 7. Conclusions

FHIR is an emerging standard with increasing adoption from institutions, organizations, and countries. By leveraging modern technologies and the experience from previous standards, FHIR is well positioned to be adopted as an international or widely implemented healthcare data standard, compatible with future developments and emerging technologies. Its resource-based approach allows for flexibility without losing its structure and it is designed to be modular, scalable, and patient-centric. From a technical perspective, it is designed for web technologies and is easy to implement, which facilitates its integration in new applications, and makes sense considering the interoperability envisioned by the IoT paradigm, where every new piece of data collected, from any device compliant with the standard, can be easily and seamlessly processed and integrated in healthcare systems. 

The implementation of FHIR within the Citizen Hub mobile application was designed to validate the mentioned data and provide cross-platform functionality, so information can be uploaded to any platform compliant with the standard, as was described in the scenarios of Section 5. The correlation of physical activity, health indicators, and rehabilitation with physical health and wellbeing enables a more holistic approach to healthcare, that considers non-communicable diseases such as musculoskeletal conditions and low-back pain as behavior-dependent. Individuals can leverage the use of wearables, medical devices, and other sensing devices to produce data that can be integrated into electronic health records (thus, including physical activity, health-related measurements, and risk factors, creating a more personal health record) and further use them to provide a more personalized healthcare service, that impacts not only the traditional care but also the perspective and empowerment of individuals to take action and have healthy lifestyles, that result in a better quality of life.

The implementation succeeded and validated the capabilities of the FHIR standard with an ease of implementation, demonstrating its benefits, not only providing a way for individuals to upload physical activity and vital sign measurements, using the standard format, but also being able to review and share information in real time or through daily activity reports.

This work resulted in a data collection methodology and followed the creation of the Citizen Hub mobile application, revolving on the integration of these elements in a proposed interoperable IoT architecture with different layers to support the envisioned functionalities and necessary compliance with existing guidelines. Novelty FHIR resources were also proposed, that allow users to collect and exchange relevant data related to MSDs, contributing to interoperability and decision-making for prevention and rehabilitation plans. This was based on a literature review of existing applications of FHIR, and it was later tested and validated in three different European Union health research projects, with data pertaining to health, wellbeing, and physical activity, related to the prevention and rehabilitation of MSDs.

However, as mentioned in the discussion section, there are some technical challenges when implementing the standard, considering the possible interpretations of the profiles, the requirements for existing systems to adopt the standard and be interoperable, and guaranteeing that the information is collected and meaningfully exchanged without conflicts.

## Figures and Tables

**Table 1:** Difference between HL7 (v2, v3, and CDA) and FHIR standards, adapted from [56].

Feature	HL7 v2	HL7 v3	HL7 CDA	FHIR
**Overview**	Widely used, older standard for data transmission between systems	Complex, highly structured data exchange standard	Markup standard for defining a structure of clinical documents	Modern data exchange standard with flexible elements and web-based approach
**Data format**	text-pipe-and-hats messages	XML-based	XML, document-oriented	JSON, XML, RDF
**Interoperability**	Limited, version-specific	Present but difficult to achieve	High for document exchange	High, flexible
**Complexity**	Low	High	Moderate	Low, implementer friendly
**Flexibility**	Low	Moderate	High for documents	High, modular
**Use case**	Admit discharge transfer patients, medical prescriptions, measurement results, etc.	CDA is the most used component of the V3 standard (for clinical documents exchange)	Clinical documents exchange between patients and caregivers	Covers common healthcare use cases, is used for exchange of information such as lab results, clinical letters, etc.
**Adoption rate**	High in legacy	Very low	Moderate	Rapidly growing
**Resources**	Work for exchange between systems within an organization and provides enterprise-level interoperability	Implementation of the common data model (RIM) is mandatory, thus complex and unprofitable	An essential component of data exchange in healthcare, but requires some training time	Appeal to web-savvy developers but may require training costs and additional time or the help of certified FHIR experts

**Table 2:** Smart4Health collected datasets by CUC.

Use Case	Description	Datasets ^1^
CUC 1	US EHR+ best practice infrastructure and research database	40,000
CUC 2	Smart4Health and cross-border EHR exchange	30,000
CUC 3	Smart4Health in therapy	1384
CUC 4	Smart4Health at workplace	23,427
CUC 5	Smart4Health at daily life and at workplace	7262
CUC 6	Smart4Health in daily life and working in community care	
CUC 7	Smart4Health at daily work in the hospital	
CUC 8	Smart4Health at travel and at daily life	34,500
Total		136,573

^1^ based on an estimation of valid datasets.

## Data Availability

The data collected in the different presented scenarios is not public.
